# Expanded repertoire of kinetoplast associated proteins and unique mitochondrial DNA arrangement of symbiont-bearing trypanosomatids

**DOI:** 10.1371/journal.pone.0187516

**Published:** 2017-11-13

**Authors:** Silvana Sant´Anna de Souza, Carolina Moura Catta-Preta, João Marcelo P. Alves, Danielle P. Cavalcanti, Marta M. G. Teixeira, Erney P. Camargo, Wanderley De Souza, Rosane Silva, Maria Cristina M. Motta

**Affiliations:** 1 Laboratório de Ultraestrutura Celular Hertha Meyer, Instituto de Biofísica Carlos Chagas Filho, Universidade Federal do Rio de Janeiro, Rio de Janeiro, Brazil; 2 Laboratório de Metabolismo Macromolecular Firmino Torres de Castro, Instituto de Biofísica Carlos Chagas Filho, Universidade Federal do Rio de Janeiro, Rio de Janeiro, Brazil; 3 Instituto Nacional de Ciência e Tecnologia em Biologia Estrutural e Bioimagens – Rio de Janeiro, Brazil; 4 Departamento de Parasitologia, Instituto de Ciências Biomédicas, Universidade de São Paulo, São Paulo, Brazil; 5 Laboratório de Microbiologia, Instituto Nacional de Metrologia, Qualidade e Tecnologia – Inmetro, Rio de Janeiro, Brazil; University of Ostrava, CZECH REPUBLIC

## Abstract

In trypanosomatids, the kinetoplast is the portion of the single mitochondrion that is connected to the basal body and contains the kDNA, a network composed by circular and interlocked DNA. The kDNA packing is conducted by Kinetoplast Associated Proteins (KAPs), which are similar to eukaryotic histone H1. In symbiont-harboring trypanosomatids (SHTs) such as *Angomonas deanei* and *Strigomonas culicis*, a ß-proteobacterium co-evolves with the host in a mutualistic relationship. The prokaryote confers nutritional benefits to the host and affects its cell structure. Atomic force microscopy showed that the topology of isolated kDNA networks is quite similar in the two SHT species. Ultrastructural analysis using high-resolution microscopy techniques revealed that the DNA fibrils are more compact in the kinetoplast region that faces the basal body and that the presence of the symbiotic bacterium does not interfere with kDNA topology. However, RT-PCR data revealed differences in the expression of KAPs in wild-type protozoa as compared to aposymbiotic cells. Immunolocalization showed that different KAPs present distinct distributions that are coincident in symbiont-bearing and in symbiont-free cells. Although KAP4 and KAP7 are shared by all trypanosomatid species, the expanded repertoire of KAPs in SHTs can be used as phylogenetic markers to distinguish different genera.

## Introduction

DNA compactness is a characteristic feature of cell evolution, being manifested from prokaryotes to eukaryotes, including viruses and organelles of symbiotic origin. Eukaryotic genomes are packed in the nucleosome, which comprises DNA and histones. Bacteria contain histone-like proteins, such as HU and H-NS, that have a generalized compacting role [1–2]. Conversely, in the mitochondrion, which has a prokaryote endosymbiotic origin, DNA is condensed by HMG-like proteins and histone H1-like proteins of eukaryotic origin [3]. These roles have been associated with the massive loss of the mitochondrial genome [4]. Thus, the compaction of mitochondrial DNA is different from the compaction of nuclear chromatin or the bacterial nucleoid [5–6].

In trypanosomatid protozoa, mitochondrial DNA packing is conducted by Kinetoplast Associated Proteins (KAPs), which are histone H1-like proteins. The kinetoplast comprises an enlarged portion of the single branched mitochondrion and contains the mitochondrial DNA, which is called kDNA. The kDNA presents one of the most complex and intriguing arrangements in nature, comprising two types of interlocked circular DNA, minicircles and maxicircles. This network contains thousands of minicircles (approximately 5,000 in *Crithidia fasciculata*), which encode small guide RNAs that are templates for editing maxicircle transcripts. Differently from other eukaryotes, mitochondrial transcripts produced from maxicircles require the addition and deletion of uridine residues to generate translatable mRNAs. Maxicircles are not as numerous as minicircles (25 to 50 in *C*. *fasciculata*) and, similar to other mitochondrial DNAs, they encode rRNAs and proteins related to ATP production [7].

KAPs have low molecular weights and are highly basic proteins rich in alanine and lysine residues, which may be involved in neutralizing the negatively charged DNA. Such proteins contain, in their amino-terminal region, a cleavable nine amino acid pre-sequence involved in protein import to the kinetoplast [8]. KAPs were characterized first in *C*. *fasciculata* as H1 histone-like proteins with structural functions [8–9]. Accordingly, the disruption of both alleles of the CfKAP1 gene resulted in kDNA rearrangement [10]. Although KAPs are mainly associated with kDNA organization, other functions related to cell growth, respiration, and mitochondrial transcription as well as kDNA replication and/or segregation have been attributed to these proteins, as demonstrated in knockout cells of *C*. *fasciculata* and *Trypanosoma brucei* for KAP genes [11–12]. In *Trypanosoma cruzi*, KAP3 null mutants did not present alterations in cell growth, morphology or kDNA topology. Parasite differentiation and infectivity were not affected either, indicating that other KAPs may compensate for TcKAP3 activity [13]. Interestingly, both KAP4 and KAP6 have distinct kinetoplast distribution patterns in the different developmental stages of *T*. *cruzi*, which have variations in kDNA topology [14].

Some trypanosomatids maintain a mutualistic relationship with a symbiotic bacterium and have been used to investigate cell division, metabolic co-evolution, and gene transfer [15–20]. Such symbiont-harboring trypanosomatids (SHTs) form a monophyletic group and are found in the following three genera: *Angomonas*, *Strigomonas* and *Kentomonas*, totaling seven species [21–22]. Recently, a novel symbiotic association between a kinetoplastid protist, *Novymonas esmeraldas*, and an endosymbiont was described, However, in this case neither the host protozoan nor the bacterium are closely related to SHT species and their respective symbionts. Interestingly, the number of symbionts per protozoan varies (between 1 and 15), and some host cells do not contain bacteria. This indicates that the association is more recent in comparison to those described for other genera of SHTs (Kostygov *et al*. 2016). Usually, when compared to other family members, symbiont-containing protozoa present ultrastructural alterations such as a reduced paraflagellar rod, a distinct cytoskeleton distribution, and an atypical kinetoplast form, which contains a looser kDNA arrangement [23–25]. To study the symbiont´s influence on morphological and physiological aspects of the trypanosomatid host, aposymbiotic (or cured) strains were generated after antibiotic treatment [26–28]. Such strains are able to grow *in vitro* in supplemented medium but are unable to colonize insects, as expected in obligatory associations where both partners only live together [29].

After the genome sequencing of two symbiont-harboring species, the following three KAPs were identified and tentatively classified: KAP 4 and KAP 3 in *Angomonas deanei* and KAP 4 and KAP 2 in *Strigomonas culicis* [30]. Previous studies showed evidence of KAP 3 and KAP4 in *Leishmania*, *Trypanosoma* and *Crithidia* genera, whereas KAP 1 and KAP 2 were only identified in *Leishmania* and *Crithidia* species. KAPs 6 and 7 were only found in heteroxenous trypanosomatids of the *Trypanosoma* and *Leishmania* genera, whereas KAP5 was only identified in *C*. *fasciculata* [8, 14].

In the present study, we showed the phylogenetic relationships, expression and localization of KAPs in SHTs for the first time. New genome searches identified novel KAPs in these trypanosomatids, which can be used as molecular markers of protists of the genera *Angomonas* and *Strigomonas*. Furthermore, an investigation of the kinetoplast by high-resolution microscopy techniques showed that the ultrastructure of wild-type (WT) and aposymbiotic (APO) cells of the same species is similar, indicating that the presence of the symbiont does not influence kDNA arrangement.

## Materials and methods

### Phylogenetic inference

For phylogenetic analysis, all previously characterized KAP protein sequences [14] were used as queries to collect the corresponding putative orthologous sequences from the full NCBI nr protein database by BLASTP searches (E-value cutoff of 1e-10). All candidate KAP protein sequences found were then searched (TBLASTN) against the genomes of trypanosomatids for which protein predictions had not been determined, and the gene sequences found were manually extracted and confirmed. All novel sequences were searched again against nr and the genomes iteratively until no new KAP sequences emerged. All analyses were performed at the protein sequence level. Sequences were aligned by Muscle v. 3.8.31 [31]. Phylogenetic inferences were performed by the Bayesian method as implemented in MrBayes v. 3.2.6 [32] using the mixed amino acid substitution model and gamma-distributed heterogeneity rates. Two parallel runs of four chains each were performed, and the analysis was run until convergence of the runs, i.e., when the average standard deviation of split frequencies became lower than 0.01. Trees were drawn and formatted using TreeGraph2 [33] and Dendroscope [34], with subsequent cosmetic adjustments performed manually with the Inkscape vector image editor (http://inkscape.org).

### Cell culture

The *Angomonas deanei* and *Strigomonas culicis* wild type (WT—ATCC 044, ATCC 30268) and aposymbiotic (APO—ATCC 30969, ATCC 30268) strains were cultured in Warren´s medium [35] supplemented with 10% fetal bovine serum. Protists were maintained by weekly passages by inoculating 10% of an established cell culture in fresh medium. Cells were grown at 28°C for 24–48 h, which corresponds to the exponential growth phase, and were then stored at 4°C.

### Quantification of KAP transcripts by quantitative PCR

Total RNA was isolated from mid log phase cells (1 x 10^8^ cells/mL) from both WT and APO *A*. *deanei* and *S*. *culicis* strains using Trizol reagent (Invitrogen, Carlsbad, USA), according to the manufacturer’s instructions. After treatment with RQ1Dnase (Promega^®^) for 1 hour at 37°C, approximately 2.5 μg of total RNA from both *A*. *deanei* and *S*. *culicis* strains were used to obtain the cDNA strand using the High Capacity cDNA Reverse Transcription Kit (Applied Biosystems^®^) according to the manufacturer's protocol. Quantitative analyses of KAP transcripts were performed using the Power SYBR Green PCR Master Mix (Fermentas^®^) kit, and reactions were performed in duplicate. Primers for qPCR are listed in S1 Table. Reactions were performed in ViiA7 Applied Biosystems^®^ equipment. Expression of KAP transcripts was determined by relative quantification comparing the values of cycle threshold (Ct) obtained for the WT and APO strains. To normalize the Ct values, GAPDH transcripts of *A*. *deanei* (AGDE12097) and *S*. *culicis* (STCU09216) were used as the endogenous controls for relative expression. Fold change expression was calculated using 2^-ΔΔCt^. Statistic analysis were calculated using the ΔCt values of three independent biological replicates. Paired t test was performed using GraphPad Prism considering as reference the WT strain.

### Production and purification of antibodies

Antibodies raised against *A*. *deanei* and *S*. *culicis* KAPs (KAP4, aKAP23 and stKAPy) were obtained from peptide sequences as previously identified in SHTs [30]. KAP polyclonal antibodies against synthetic peptides were produced in rabbits by EZBiolabs^®^ (http://www.ezbiolab.com/monoclonal.html) based on their immunogenic power: *A*. *deanei* KAP4 (PAKEKKEKKAKAA), *S*. *culicis* KAP 4 (QLSPHEKQKLDVR), aKAP23 (PTKKSVKPTKKSI) and stKAPy (VAKKLKVASKLSK). These lyophilized samples were resuspended in double distilled water according to the manufacturer's recommendations and was then subjected to affinity purification on nitrocellulose membranes. Then, 420 μg of each synthetic peptide was transferred to a nitrocellulose membrane, which was treated with blocking solution (PBS, pH 7.5, with 3% BSA) for 1 hour at room temperature. Subsequently, antibodies were incubated with the membrane for 72 hours at 4°C and then washed with PBS, pH 7.5. Elution was performed with a solution containing 0.2 M glycine and 1 mM EDTA, pH 4.0 for 8 min and subsequently neutralized with a pre-chilled solution of 10 mM Tris until reaching a pH of 7.0. The same procedure was adopted with the pre-immune rabbit serum.

### Western blot analysis

Antibody specificity was assessed in wild type and aposymbiotic strains of *A*. *deanei* and *S*. *culicis* by Western blotting. Each lane was loaded with 5 x10^6^ cells resuspended in Laemmli buffer of a 4–14% NuPAGE Bis-Tris gel (Invitrogren) in MES running buffer and transferred to Nybond-C nitrocellulose membranes (GE Healthcare). The molecular weights were estimated by comparing sizes with Protein Marker Extended PS13 (GeneON). Following the transfer of the proteins to nitrocellulose, membranes were blocked with 3% non-fat milk in TBST. Then, membranes were incubated overnight at 4°C with the blocking solution containing primary antibodies produced against KAPs that were produced as described above. Antibodies were used at different concentrations to detect their specific targets, as follows: AdKAP4, 1:2000; AdKAP23, 1:500; ScKAP4 and stKAPy, 1:250. Membranes were washed three times in TBST before incubation with horse radish peroxidase (HRP)-conjugated secondary rabbit antibody at 1:5,000 dilution for 1 h at room temperature. Control assays were performed with each of the pre-immune IgG. MAb anti-α-tubulin (1:1000) was used as an internal loading control (EP1332Y from Abcam, Cambridge, U.K.) and revealed after incubation with horse radish peroxidase (HRP)-conjugated secondary mouse antibody at 1:5,000 dilution. After washing in TBST, the membrane was treated with Clarity Max Western ECL Blotting Substrate (Bio Rad) according to manufacturer's instructions. Images were acquired using ChemiDoc MP Imaging system (Bio Rad).

### Immunofluorescence

Protozoa were washed in PBS, pH 8.0, and fixed with freshly prepared 1% formaldehyde in PBS for 1 h. After fixation, the cells were deposited on poly-L-lysine-coated glass slides and permeabilized for 30 min with 2% Nonidet P-40 (NP-40) diluted in PBS. The slides were incubated in blocking solution containing 1.5% bovine serum albumin (BSA), 0.5% teleostean gelatin (Sigma Aldrich), and 0.02% Tween 20 in PBS. Next, slides were incubated for 1 h with antibodies against the symbiont’s porin [36] diluted 1:5 in blocking solution. Antibodies produced against KAPs of *A*. *deanei* (KAP4 and aKAP23) and *S*. *culicis* (KAP4 and stKAPy) were diluted 1:50 in blocking solution. After incubation with primary antibodies, cells were washed and incubated for 45 min with Alexa 488-conjugated anti-mouse IgG and Alexa 546-conjugated anti-rabbit IgG (Molecular Probes, USA) diluted in blocking solution to the final concentration of 3 mg∕mL. Samples incubated with pre-immune sera or not incubated with the primary antibodies were used as negative controls. Slides were mounted using the antifade reagent ProLong Gold containing 5 mg∕mL DAPI (4′,6-diamidino-2-phenylindole) (Molecular Probes). Serial image stacks (0.2-μm Z-increment) were collected at 100x (oil immersion 1.3 NA) on a motorized AxioVision 4.8 microscope (Zeiss, Germany). All images were collected and processed using Zen software (Zeiss, Germany).

### Transmission electron microscopy (TEM)

For routine TEM, protozoa were fixed for 1 h in 2.5% glutaraldehyde diluted in 0.1 M cacodylate buffer, pH 7.2. Next, cells were washed twice with the same buffer and post-fixed in a 1% OsO_4_, 0.8% KFe(CN)_6_, 5 mM CaCl_2_ solution diluted in 0.1 M cacodylate buffer. Cells were then washed, dehydrated in a graded series of acetone solutions, and embedded in Epon. Ultrathin sections were stained with uranyl acetate and lead citrate before observation using a Tecnai Spirit (FEI Company, Netherlands) transmission electron microscope.

### Electron tomography

Conventional electron microscopy was used for kinetoplast analysis. Sections of 200 nm were collected on Formvar-coated slot copper grids. Samples were post-stained with uranyl acetate and lead citrate, incubated with 10 nm colloidal gold on both sides for 5 min and then washed in distilled water. Sections were observed using a 200-kV transmission electron microscope Tecnai G2 (FEI Company, Netherlands) equipped with a 4k x 4k CCD camera (Eagle, FEI Company), which was used to record the tomograms. Tilt series from + 60° to—60° with an angular increment of 1° were used to acquire all tomograms.

### Atomic force microscopy (AFM)

kDNA networks of *A*. *deanei* and *S*. *culicis* were isolated according to the protocol developed by [37]. For AFM analysis, the solutions containing the isolated kDNA networks (50–100 ng.ml^-1^) were mixed with 25 mM magnesium chloride and then placed on a freshly cleaved mica surface, as described by Cavalcanti et al. [38]. Samples were incubated at room temperature, rinsed gently in Milli-Q water and critical point-dried in a Leica EM CPD030 apparatus. The images of networks were acquired with a Bioscope Catalyst atomic force microscope (Bruker) in air using tapping mode. The microscope was mounted with a rectangular tip working in a nominal resonant frequency of approximately 75 kHz. Images were processed with Nanoscope analysis software.

## Results

### Phylogenetic analyses of KAPs

**Fig 1:**
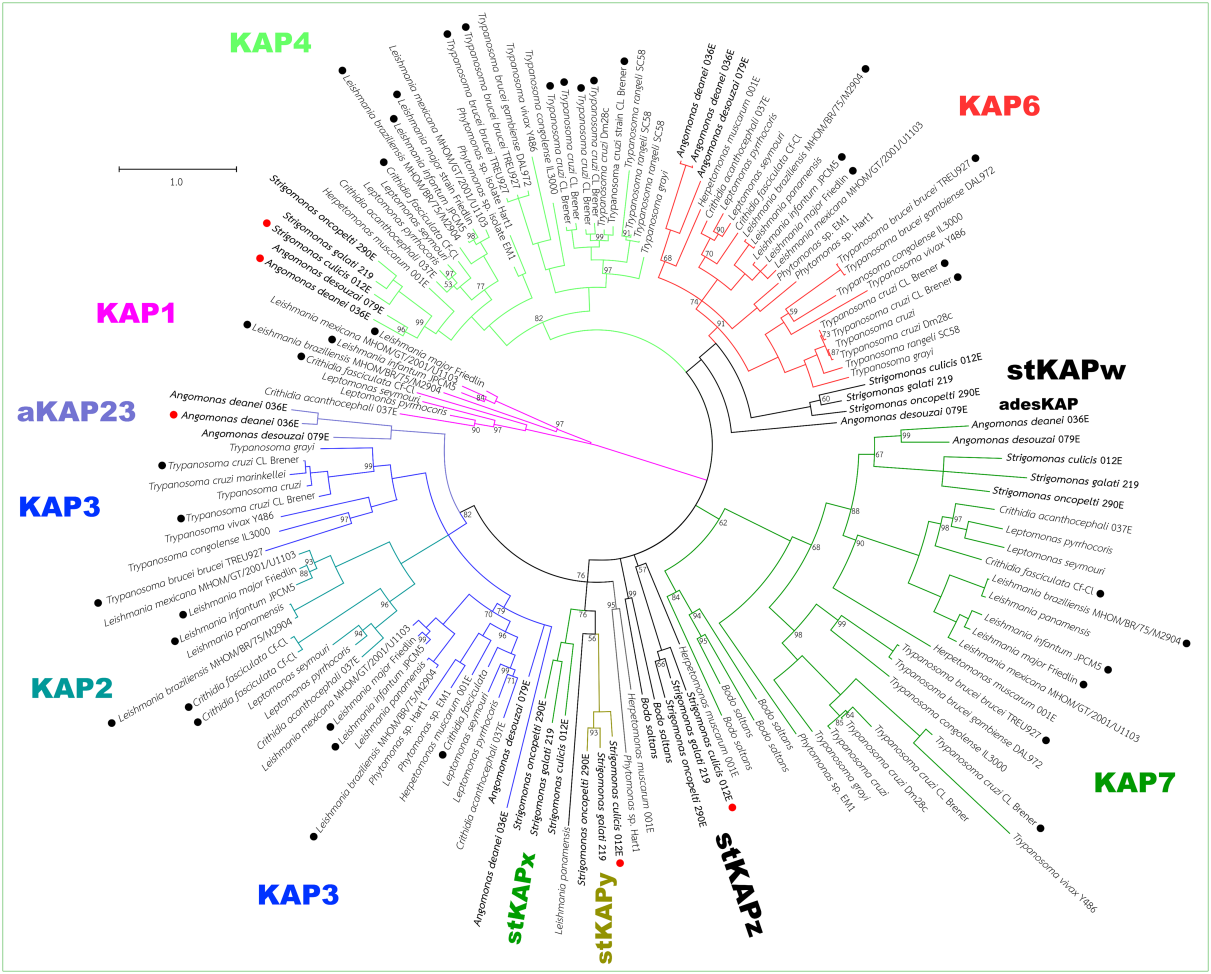
Phylogenetic relationships among KAPs as determined by Bayesian inference. Branches are colored according to the putative KAP type identified. Numbers on nodes represent the Bayesian posterior probability for the corresponding clade (clades without numbers are those presenting 100% posterior probability). Black circles next to taxon names indicate sequences used by Cavalcanti et al. 2009 [14]; red circles indicate sequences used by Motta et al. 2013 [30].

Increasing availability of trypanosomatid genome sequences enabled the investigation of Kinetoplast Associated Proteins (KAPs). A collection of 240 sequences coding for different KAPs from genera *Trypanosoma*, *Leishmania*, *Phytomonas*, *Crithidia*, *Herpetomonas*, *Angomonas*, *Strigomonas*, *Leptomonas*, and *Bodo* were analyzed by phylogenetic inference. This analysis indicated that at least seven types of KAPs were grouped according to the corresponding support values (Fig 1; sequences used in [30] are marked by red dots).

KAP1 sequences were identified in *Leishmania*, *Crithidia* and *Leptomonas*, forming a well-supported clade distant from the other KAPs. Genes for KAP4 and KAP7 were detected in all trypanosomatids and KAP6 was found in most genera that comprise this family, but not in *Strigomonas*. However, KAP2 and KAP3 formed one single well-supported clade considering Bayesian Inference Posterior Probability (BIPP) of 82%.

In this work, the analysis of SHT sequences previously thought to be coding for KAP2 and KAP3 showed that they did not group with other previously known KAPs, suggesting that they probably represent new KAP types. The *Angomonas deanei* sequence, previously identified as a KAP3 [30], was not grouped with the clade comprising *Leishmania*, *Crithidia* and *Leptomonas*, as would be expected, considering the currently accepted Trypanosomatidae phylogeny. Instead, this *Angomonas* KAP was placed as an outgroup to the large clade comprising all KAP2 and KAP3 sequences (BIPP of 82%); this sequence belongs to a small group, which is named aKAP23 in this study, containing only *Angomonas* sequences. On the other hand, we have identified new *Angomonas* KAP sequences, one for *A*. *deanei* and another for *A*. *desouzai*. These genes should be considered as part of the KAP3 type, since they group within that clade with moderate BIPP (79%), although the grouping is not well-resolved (Fig 1).

The KAP2 of *Strigomonas culicis* was placed far from all KAP2 sequences previously identified and are present exclusively in genera *Leishmania*, *Crithidia*, and *Leptomonas*. Here, this and other closely related *Strigomonas* sequences were placed as an outgroup of the large clade containing all KAP2 and KAP3 sequences plus aKAP23 (Fig 1). Thus, they might comprise a novel KAP type named stKAPy, which currently forms a monophyletic taxon with another *Strigomonas* orthologous group (stKAPx) as well as one gene from *Leishmania panamensis* (all grouping with moderate BIPP support of 76%).

*Strigomonas* presents two deep-branching clades and *Angomonas desouzai* presents one. One of the novel *Strigomonas* KAP types (stKAPw) as well as the *A*. *desouzai* type (adesKAP) are closer to the KAP6 type, clustering with a BIPP of 100% in our analyses. However, the two genera do not form a monophyletic group since *A*. *desouzai* branches off at the base of the group and separately from the *Strigomonas* genes. It is dubious to consider these genes as part of the KAP6 type, since their phylogeny strongly differs from the currently known species phylogeny for the SHTs.

It is interesting to note that the KAP6 genes from *Angomonas deanei* (two non-identical copies) and *A*. *desouzai* are present in their expected places in the tree, near *Leishmania*, *Crithidia*, and *Leptomonas* species. *Strigomonas* species, on the other hand, do not present such genes, indicating that they have lost their copy of KAP6.

The other deep-branching *Strigomonas* KAP type (stKAPz), which also includes one *Herpetomonas muscarum* sequence (although with the low BIPP of 57%), is related to most other KAP types at the basal polytomy that separates all KAP types. Some of the genes identified here could not be confidently and unambiguously placed in any of the currently existing KAP types, such as the six *Bodo saltans* genes. Such sequences form two clades that branch very basally in the tree: one at a polytomy that involves nearly all KAP types, and another that groups with low BIPP (62%) in the KAP7 clade. The repertoire of KAPs present in trypanosomatid protozoa is listed in S2 Table.

### Quantification of KAP transcripts by quantitative PCR

**Fig 2:**
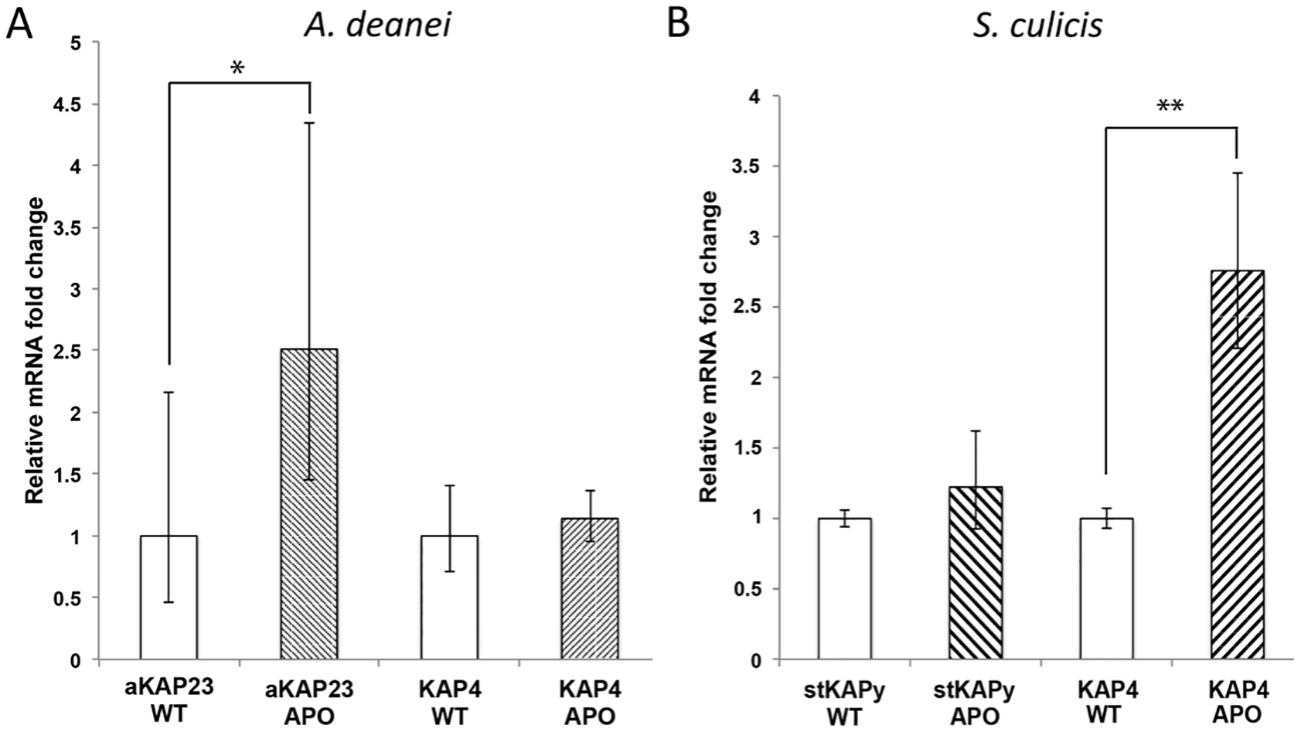
Quantification of KAP transcripts for KAP4, aKAP23 and stKAPy by RT-qPCR. The relative amount of KAPs was estimated by comparison with the GAPDH expression of WT trypanosomatids. (A) *A*. *deanei* KAPs, (B) *S*. *culicis* KAPs. APO, aposymbiotic strain; WT, wild type strain. AdKAP4—p > 0.05, aKAP23—p < 0.05 (*), stKAPy—p > 0.05, ScKAP4—p < 0.01 (**).

Here, we also evaluated KAP expression in the best studied SHTs, *Angomonas deanei* and *Strigomonas sulicis*, and their aposymbiotic (APO) counterparts (Fig 2). Quantification assays of transcripts for KAP4 from *A*. *deanei* and *S*. *culicis*, aKAP23 from *A*. *deanei* and stKAPy from *S*. *culicis* were performed by RT-qPCR. Such sequences were chosen considering our present phylogenetic study, which postulates that the *kap4* gene is present in all trypanosomatids analyzed so far, whereas the *akap23* and *stkapy* genes were only found in the *Angomonas* and *Strigomonas* genera, respectively. The results showed that in *A*. *deanei* the *kap*4 gene (Ad*kap*4) expression was similar in both strains, while the number of transcripts for aKAP23 was 2.5 times higher in the APO strain (RQ = 2.512) (Fig 2A). In the APO strain of *S*. *culicis*, transcripts for KAP4 increased almost 3 times when compared to the WT cells (RQ = 2.761), while the *stkapy* gene showed similar expression in both strains of this species. It means that only in aposymbiotic strains, aKAP23 of *A*. *deanei* and KAP4 of *S*. *culicis* presented a significant augment in expression (Fig 2B).

### Western blot analysis of KAPs in symbiont-bearing trypanosomatids

Polyclonal antibodies raised against different synthetic KAP synthetic peptides were probed against cell lysates. The affinity-purified IgG antibodies identified the following proteins of *A*. *deanei* and *S*. *culicis*: AdKAP4 detected a single peptide of 13 kDa, AdKAP23 a 16 kDa peptide, ScKAP4 a 14 kDa peptide and stKAPy a 17 kDa peptide. Controls performed with each of the pre-immune IgG did not recognize any protein. MAb anti-α-tubulin was used as an internal loading control (S1 Fig).

### KAP immunolocalization

**Fig 3:**
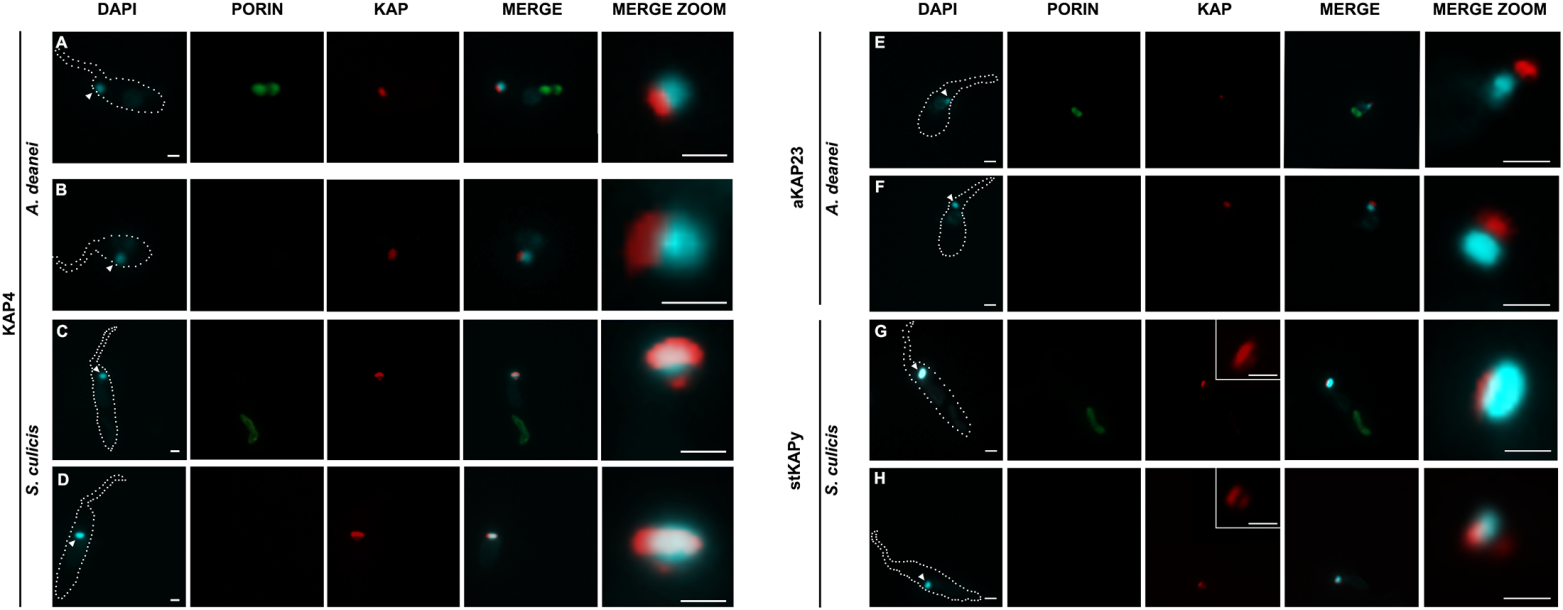
Immunolocalization of KAPs on WT and APO strains of *S*. *culicis* and *A*. *deanei*. DAPI was used to label the nucleus and the kinetoplast, and the anti-porin antibody to identify the symbiotic bacterium in WT cells. (A-B): The anti-KAP4 antibody labeled the kinetoplast region that faces the anterior end of *A*. *deanei* in WT (A) and APO (B) cells. Note that this labeling in part coincides with DAPI staining. (C-D): For *S*. *culicis*, KAP4 labeling is dispersed through the kinetoplast of both strains and overlaps those for DAPI. (E-F): The anti-aKAP23 antibody generated a similar and specific labeling for WT (E) and APO (F) cells of *A*. *deanei*. In this case, an overlap with DAPI was not observed indicating that aKAP23 does not co-localize with the kDNA and is probably located at the KFZ. (G-H) Regarding stKAPy, this protein also faces the anterior part of the cell body in both strains, however the labeling is more disperse when compared to that obtained for aKAP23. Arrowheads indicate the kinetoplast. Scale bars equal to 1 μm.

To further investigate the distribution of these proteins, we performed immunofluorescence analyses by using antibodies generated against stKAPy, aKAP23, and KAP4 in WT and APO strains of *A*. *deanei* and *S*. *culicis* (Fig 3). The labeling of KAP4 in WT and APO strains of *A*. *deanei* is mainly observed in the region facing the anterior end of the protozoan and in part coincides with DAPI staining (Fig 3A and 3B). In the case of *S*. *culicis*, KAP4 labeling is seen spread through the kinetoplast of both strains and overlaps those for DAPI (Fig 3C and 3D). Considering aKAP23, labeling is not coincident with DAPI staining. This protein is localized in the kinetoplast region that faces the anterior end of the protozoan cell body, perhaps at the kinetoflagellar zone (KFZ) (Fig 3E and 3F). Regarding stKAPy, this protein also faces the anterior part of the cell body. However, differently from aKAP23, its labeling is more disperse (Fig 3G and 3H).

### Kinetoplast ultrastructure of symbiont-bearing trypanosomatids

**Fig 4:**
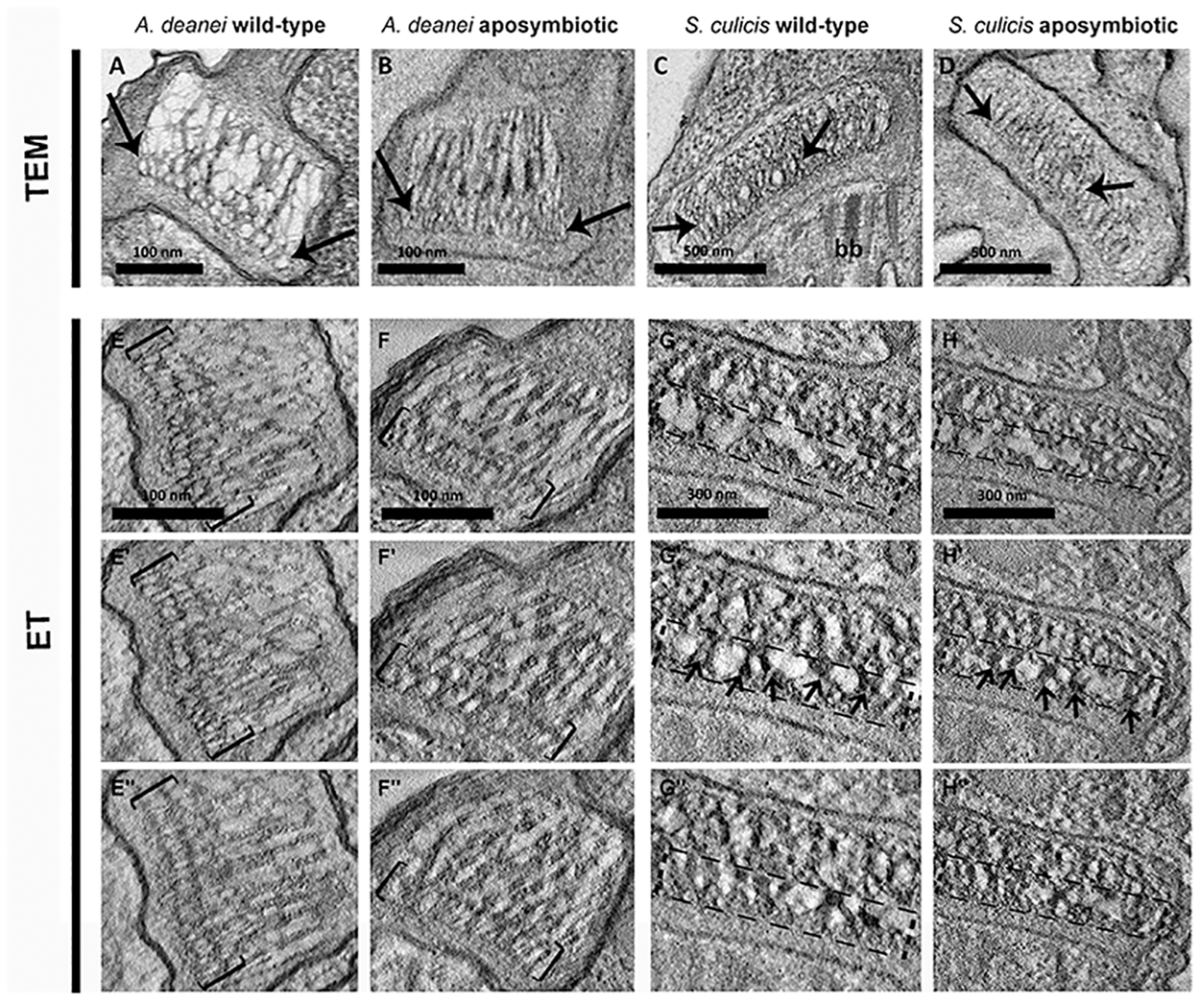
Kinetoplast ultrastructure of WT and APO strains of *A*. *deanei* and *S*. *culicis*. A-D: Kinetoplast ultrastructure observed by transmission electron microscopy (TEM). A-B: Both strains of *A*. *deanei* present a similar kinetoplast ultrastructure that presents a trapezoid shape and a looser arrangement of the central area in relation to the region that faces the basal body (bb) where kDNA is more condensed (black arrows). C-D: In WT and APO strains of *S*. *culicis*, the kinetoplast displays an arch shape and the more condensed layer close to the basal body (black arrows). E-H: Kinetoplast ultrastructure observed by electron tomography (ET). E-F: This high-resolution technique confirmed that both *A*. *deanei* strains have a similar kDNA network topology presenting the densely packed kDNA region facing the basal body (brackets). (E, F) top, (E’, F’) middle and (E”, F”) bottom regions of the kinetoplast as visualized by electron tomography. G-H: In both strains of *S*. *culicis*, the kinetoplast is flatter when compared to that found in *A*. *deanei*, and the compact DNA fibers occupy approximately half of the kDNA network (dashed rectangle). (G, H) top, (G’, H’) middle and (G”, H”) bottom regions of the kinetoplast as visualized by electron tomography. In G’ and H’ arrows indicate the condensed kDNA fibers.

Considering that the different anti-KAP antibodies labeled the symbiont-containing and symbiont-free cells similarly, we decided to compare the kinetoplast ultrastructure of WT and APO strains of *A*. *deanei* and *S*. *culicis* by electron microscopy techniques (Fig 4). First, routine transmission microscopy showed that kDNA topology arrangement is maintained in both species, even in the absence of the symbiont. In *A*. *deanei*, the kDNA fibers are disposed in a looser arrangement inside the trapezoid-shaped kinetoplast (Fig 4A and 4B), a hallmark of the *Angomonas* genus. Similarly, in *S*. *culicis*, the kDNA presents a looser array, but in this case, it is contained in an arch-shaped kinetoplast (Fig 4C and 4D). A more densely packed array of kDNA fibers appears to be present in the network region that is closer to the basal body (Fig 4A–4D arrows).

The use of a high-resolution technique, electron tomography, confirmed that WT and APO strains of *A*. *deanei* and *S*. *culicis* present a looser kDNA arrangement in the central area of the kinetoplast. The kDNA fibers are packed within the kinetoplast, perpendicular to the axis of the flagellum. Another interesting ultrastructural aspect was revealed by this technique: the kDNA presents a thicker layer composed of densely packed fibers in the region that faces the basal body in both strains of *S*. *culicis* and *A*. *deanei* (Fig 4E–4H, S1 and S2 Videos). The total surface area of the *A*. *deanei* kinetoplast is 2.1 μm^2^ ± 150 nm^2^ (n = 6; ± s.d.), and the area containing densely packed kDNA closer to the basal body is approximately 40 nm^2^. In *S*. *culicis*, this area is approximately 57 nm^2^ and the kinetoplast area corresponds to 3.4 μm^2^ ± 120 nm^2^ (n = 6, ± s.d.).

**Fig 5:**
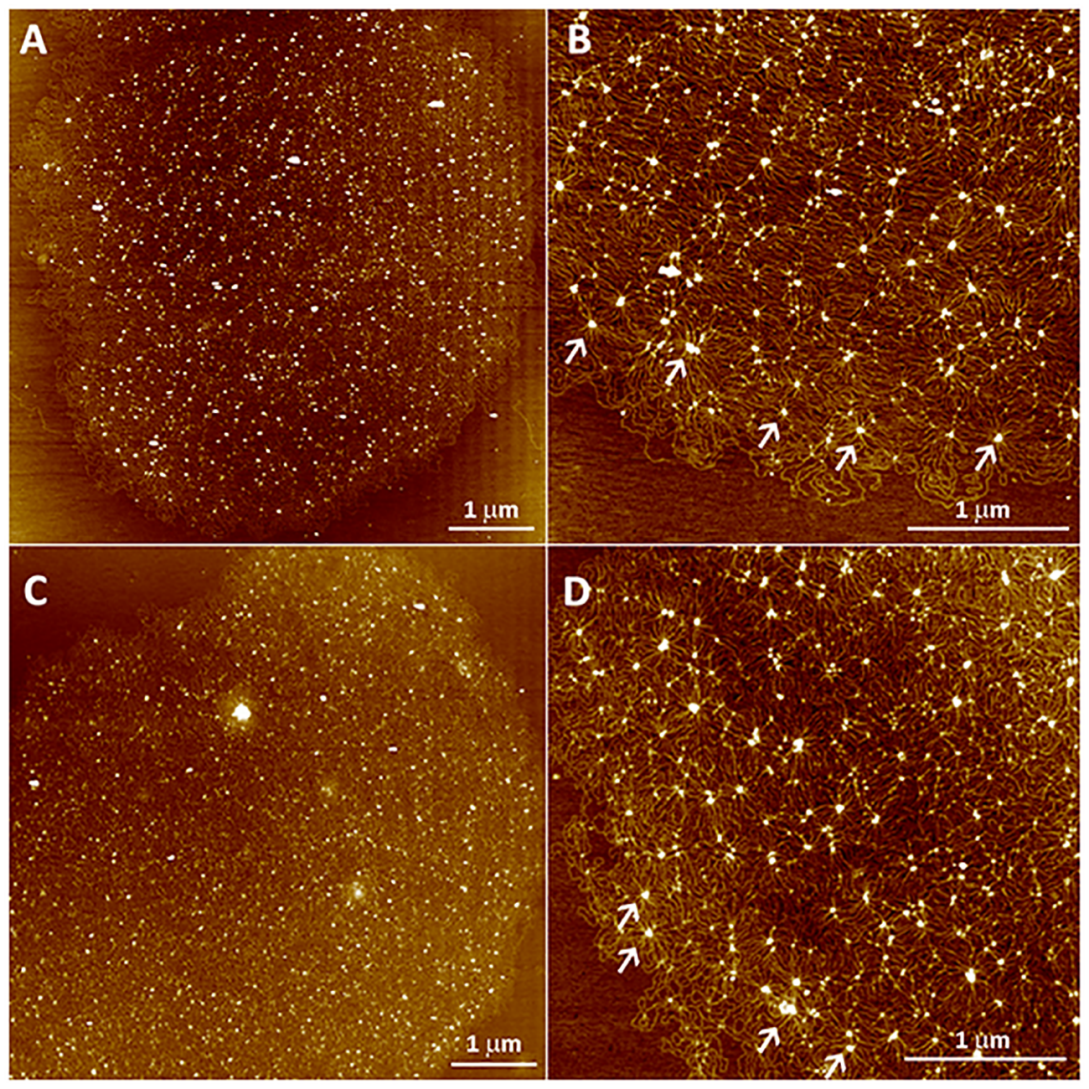
AFM analysis of isolated kDNA networks of *A*. *deanei* (A-B) and *S*. *culicis* (C-D). Both species present an arrangement composed of circles uniformly distributed throughout a massive network of kDNA molecules. Clusters of DNA forming rosette-like structures (B and D, arrows) were seen along the network and correspond to regions where the DNA molecules crossover themselves.

To complement the information acquired by electron microscopy and observe the kDNA arrangements of SHTs in more detail, networks of both species were isolated and analyzed using atomic force microscopy (AFM). Since the kinetoplast ultrastructure of WT and APO strains was quite similar, we analyzed only the networks isolated from wild type species with the aim of comparing the differences and/or similarities among them by AFM. The data revealed that the intact kDNA is composed of interlocked circular DNA molecules (Fig 5) forming networks with sizes of 7.34 x 6.47 ± 0.1 x 0.3 μm for *A*. *deanei* and 7.43 x 6.34 ± 1.1 x 0.8 μm for *S*. *culicis*. The heights of kDNA networks varied from 0.4 to 8 nm in *A*. *deanei* and 0.5 to 8 nm in *S*. *culicis*, indicating that in both species kDNA molecules crossover themselves about 20 times. The kDNA presented a similar structure when the two SHT species were compared and numerous clusters of DNA molecules forming rosette-like structures were seen along the network (Fig 5B and 5D, arrows). In addition, the global pattern of the distribution of DNA molecules was similar among *Angomonas* and *Strigomonas* species. Areas presenting a differentiated topology or compaction were not observed throughout the kDNA isolated from these protozoa (Fig 5A–5D).

## Discussion

Kinetoplast associated proteins (KAPs) are small, highly basic proteins that contain lysine- and alanine-rich domains. Such proteins influence kDNA organization since they may promote the association of individual strands of DNA by charge neutralization [9, 10, 39]. KAPs play an important role on the kDNA structural organization and participate in macromolecular processes such as kDNA replication, transcription, RNA processing and translation, thus regulating minicircle and maxicircle copy numbers [12]. Although KAPs play essential roles in trypanosomatids, they have not been thoroughly characterized, especially from a phylogenetic point of view.

It is well known that effective sampling is essential for phylogenetic inference [40]. In this work, phylogenetic analyses are in accordance with previous work [14] and show that KAP types form monophyletic groups with very high Bayesian inference posterior probability (BIPP) support values. Here, we show that the most significant exception comprises the KAP2 and KAP3 types, which form one single well-supported clade (BIPP: 82%). The KAP2 genes branch in a trichotomy at the base of this clade, thus effectively making the KAP3 clade paraphyletic. This difference in tree topology compared to previous results is very likely due to sampling effects, given that, herein, a large number of additional sequences that have recently become available were now added to the analysis.

A previous report identified a KAP2 and a KAP-like sequence in the *S*. *culicis* genome as well as one KAP3 sequence in *A*. *deanei*, whereas KAP4 was present in both symbiont-bearing trypanosomatids [30]. However, in the present work, analysis of the putative KAP2 and KAP3 sequences indicated that such genes should not be included in any of the KAP types previously described [14] but should probably be part of new types instead. It is therefore uncertain whether *Angomonas* possesses the KAP3 type at all, and further experiments, particularly function-elucidating ones, are necessary to determine the function of these new genes in the kinetoplast.

In our analyses, genes for the KAP1 type seem to be present only in organisms from the *Leishmania*, *Crithidia* and *Leptomonas* genera. According to our study, it would be more parsimonious to suppose that the common ancestor of these trypanosomatids has acquired the KAP1 sequence independently. However, the potential source of this gene is unclear, since similar searches against the NCBI nr protein database do not yield robust results. Alternatively, we can speculate that the common ancestor to all Trypanosomatidae already possessed the KAP1 type, and then all clades, except those containing these three genera, have lost it over time. Another possibility is that the KAP1 type derived from other KAP types, but as its sequence has evolved rapidly enough, it is now impossible to determine its closest neighbor with confidence.

Genes for the KAP4 and KAP7 types are present in all symbiont-harboring trypanosomatids, plus the *Leishmania*, *Trypanosoma*, *Crithidia* and *Leptomonas* genera, indicating that these two KAP types are universal among trypanosomatids. Their internal phylogenetic patterns are overall consistent with the currently accepted phylogeny of the family, with the Strigomonadinae more closely related to the clade comprising *Leishmania*, *Crithidia* and *Leptomonas* and the *Trypanosoma* genus as an outgroup of these two subfamilies. This suggests that the KAP4 and KAP7 types might be of central importance for kDNA organization and mitochondrial physiology in the Trypanosomatidae family.

It is important to note that further investigation into trypanosomatid diversity could uncover new KAP types as well as identify previously known types from more genera and species, which could still change the topology of the KAP genes phylogenetic tree. Interestingly, quantification assays of KAP transcripts by RT-qPCR indicated a different expression level of these proteins when comparing trypanosomatids either containing or not containing the symbiotic bacterium. The exact nature and function of these novel putative KAPs remain to be unveiled and necessitate new studies that are stimulated by the recent discovery that the RNA interference system is active in *A*. *deanei* [41].

In the present work, immunolocalization assays using antibodies raised against KAP4, stKAPy and aKAP23 showed that all these proteins are located in the kinetoplast but show different distributions. These results indicate that each KAP may play distinct roles. KAP4 labeling, for example, coincides with DAPI staining at some points, thus indicating that this protein may be directly bound to the network. Considering that sequences for this protein are present in all trypanosomatid genomes analyzed so far, it may play an essential role, as in kDNA topology. KAP4 distribution varied according to the developmental stages of *T*. *cruzi*, which present distinct kDNA arrangements: in epimastigotes and amastigotes, the replicative forms, TcKAP4 is homogeneously distributed in the kDNA network, whereas in trypomastigotes, the infective form, such proteins is more concentrated in the kinetoplast periphery [14].

The aKAP23 protein is located in the kinetoplast region that faces the basal body, perhaps at the KFZ region, where the replication of free minicircles initiates. The stKAPy labeling is also seen in this region, however it presents a more disperse distribution in relation to aKAP23, indicating that it can be not only involved in kDNA replication but also in the redistribution of newly duplicated circles. Ultrastructural cytochemistry assays showed that basic proteins are mainly distributed in the periphery of the kDNA and in regions where the network is more packed [25, 42, 43]. Future studies focusing on the interference of gene expression can provide us with new insights into the role of KAPs in symbiont-bearing trypanosomatids.

After analyzing KAP expression levels and distribution, we performed ultrastructural studies using electron microscopy techniques. Camargo & Freymuller (1981) were the first to describe the kinetoplast of symbiont-bearing trypanosomatids. At that time, they realized that such protozoa presented a looser arrangement of kDNA, when compared to other trypanosomatid species and also observed that this characteristic was maintained in aposymbiotic strains [23]. In fact, symbiont-harboring trypanosomatids have an atypical kinetoplast format and kDNA arrangement when compared to the disk-shaped (or bar-shaped) kinetoplasts from *C*. *fasciculata* and *T*. *brucei* [7]. In the present work, we reaffirm through the use of electronic tomography that the kinetoplast shape is different in the *Angomonas* and *Strigomonas* genera, as previously proposed using other microscopy methods, such as ultrastructural cytochemistry and immunocytochemistry [25]. The first genus has a trapezoid shape, whereas the second one has an arc shape, being wider at the center than at the extremities [21, 23, 25]. In *Strigomonas*, the kDNA disk exhibits variable degrees of thickness, as evaluated by transmission electron microscopy, presenting higher values for *S*. *oncopelti* and *S*. *culicis* in relation to *S*. *galati*. In *A*. *deanei*, the average kDNA disk thickness is 400 ± 22 nm (376–419 nm) [21].

The use of this high-resolution technique revealed that in *A*. *deanei* and *S*. *culicis* the kDNA network contains a thicker electron-dense layer of fibers that face the basal body region. Such an arrangement was observed in WT and APO cells of both species and had never been described in trypanosomatids. It is also important to note that the presence of the endosymbiont does not interfere with kDNA arrangement, since the WT and APO cells from the same species present similar network topologies, as previously reported by conventional transmission electron microscopy [23].

Considering data obtained by atomic force microscopy, kDNA isolated from *A*. *deanei* and *S*. *culicis* showed similar distributions and presented network size and height equivalent to those reported for *C*. *fasciculata* [38]. In symbiont-containing species, kDNA overlapping regions correspond to rosette-like structures that were previously described by transmission electron microscopy analysis in other trypanosomatids [37]. Furthermore, we did not observe areas, in AFM images, with a differential organization of minicircles, which could correspond to the regions of higher compaction found close to the basal body, as observed by electron microscopy. This observation suggests that the formation of regions containing densely packed DNA fibers within the three-dimensional structure of the kinetoplast is an event driven by a spatial condition instead of being caused by the distinct bidimensional structural organization of minicircles. Another possibility is that the more densely packed region of the network observed in the kinetoplast results from the kDNA interaction with the Tripartite Attachment Complex (TAC). This complex is responsible for kinetoplast positioning and segregation and is constituted by a set of cytoplasmic filaments, a zone of differentiated outer and inner mitochondrial membranes, and another set of intramitochondrial filaments, forming a system that physically connects the kinetoplast and the flagellar basal body [44]. Recently, it was demonstrated that TAC102, that is located between the kDNA network and flagellum basal body, is essential for proper kDNA segregation during *T*. *brucei* cell cycle. RNAi targeting this protein generated cells with atypical kDNA arrangements and even akinetoplast protozoa [45].

When joining different microscopy methods in this work, it becomes possible to understand, at least in part, the arrangement of kDNA. Analyses by AFM showed that the sizes of the kDNA networks isolated from *A*. *deanei* and *S*. *culicis* are quite similar, whereas measurement data, as well as images obtained by electron tomography, revealed that kinetoplasts present different shapes and sizes in these two species. This means that networks must be highly packed to fit the kinetoplast area, especially in *A*. *deanei*, where this structure presents reduced dimensions compared to those of *S*. *culicis*. This may explain, at least in part, the data obtained from electron tomography of *A*. *deanei*, showing that the area of densely packed kDNA fibers facing the basal body is approximately two times larger than the area observed in *S*. *culicis*.

Considering the presence of KAPs in different trypanosomatid species, we suggest that KAP4 and KAP7 are universal and that KAP types aKAP23, stKAPx and stKAPy can be used to distinguish protists from the *Angomonas* and *Strigomonas* genera. Although genes for KAPs presented distinct expression levels in WT and APO cells, both in *A*. *deanei* and *S*. *culicis*, it was interesting to note that these differences were not reflected in the topological arrangement of kDNA in considering strains of the same species. This result indicates that the KAPs characterized here possess not only a structural role, as shown after *C*. *fasciculata* KAP1 disruption [10], but would also be involved in other cellular events, such as kDNA replication, mitochondrial mRNA transcription, and respiration [11,12]. Our previous studies showed that the presence of the symbiont influences oxygen consumption and the inhibition of the respiratory chain after using specific compounds for protein complexes [46, 47].

The kinetoplast has unique characteristics regarding the arrangement and replication of DNA, which, in itself, already makes this structure an important study model [7]. Furthermore, the kinetoplast is a well-known structure that represents a potential chemotherapeutic target in trypanosomatids [48]. In this work, we showed that trypanosomatids of the *Angomonas* and *Strigomonas* genera present the broadest repertoire of KAPs among trypanosomatids. The set of KAPs and kDNA topology are different for *A*. *deanei* and *S*. *culicis*, species that belong to genera that are phylogenetically distant but pertain to the same clade. All the new data introduced here contributes to clarifying some important aspects of the molecular and structural biology of symbiont-bearing trypanosomatids and introduces new perspectives to study the role of KAPs on kDNA topology and replication in these cells.

## Supporting information

S1 Fig Western blot analysis to test the specificity of KAP antibodies in cell extracts of WT and APO strains of *A*. *deanei* e *S*. *culicis*. Pre-immune IgG (top, controls); affinity purified IgG antibodies identifying each of the cognate KAP protein (center); Mab antibody to α-tubulin (bottom).
(TIF)

S1 Table Primers used in RT-qPCR analyses to quantify KAP transcripts in WT and APO strains of *A*. *deanei* e *S*. *culicis*. (DOC)

S2 Table Taxonomic distribution of putative KAP types in trypanosomatids. (DOC)

S1 Video Movie of the 3D reconstruction of *A*. *deanei* wild strain kinetoplast by electron tomography. (AVI)

S2 Video Movie of the 3D reconstruction of *S*. *culicis* wild strain kinetoplast by electron tomography. (AVI)
