# Supplementary figures and images for: Expanded repertoire of kinetoplast associated proteins and unique mitochondrial DNA arrangement of symbiont-bearing trypanosomatids
Source: PLoS One. 2017 Nov 13;12(11):e0187516. doi: 10.1371/journal.pone.0187516 (PMC5683618; doi:10.1371/journal.pone.0187516)

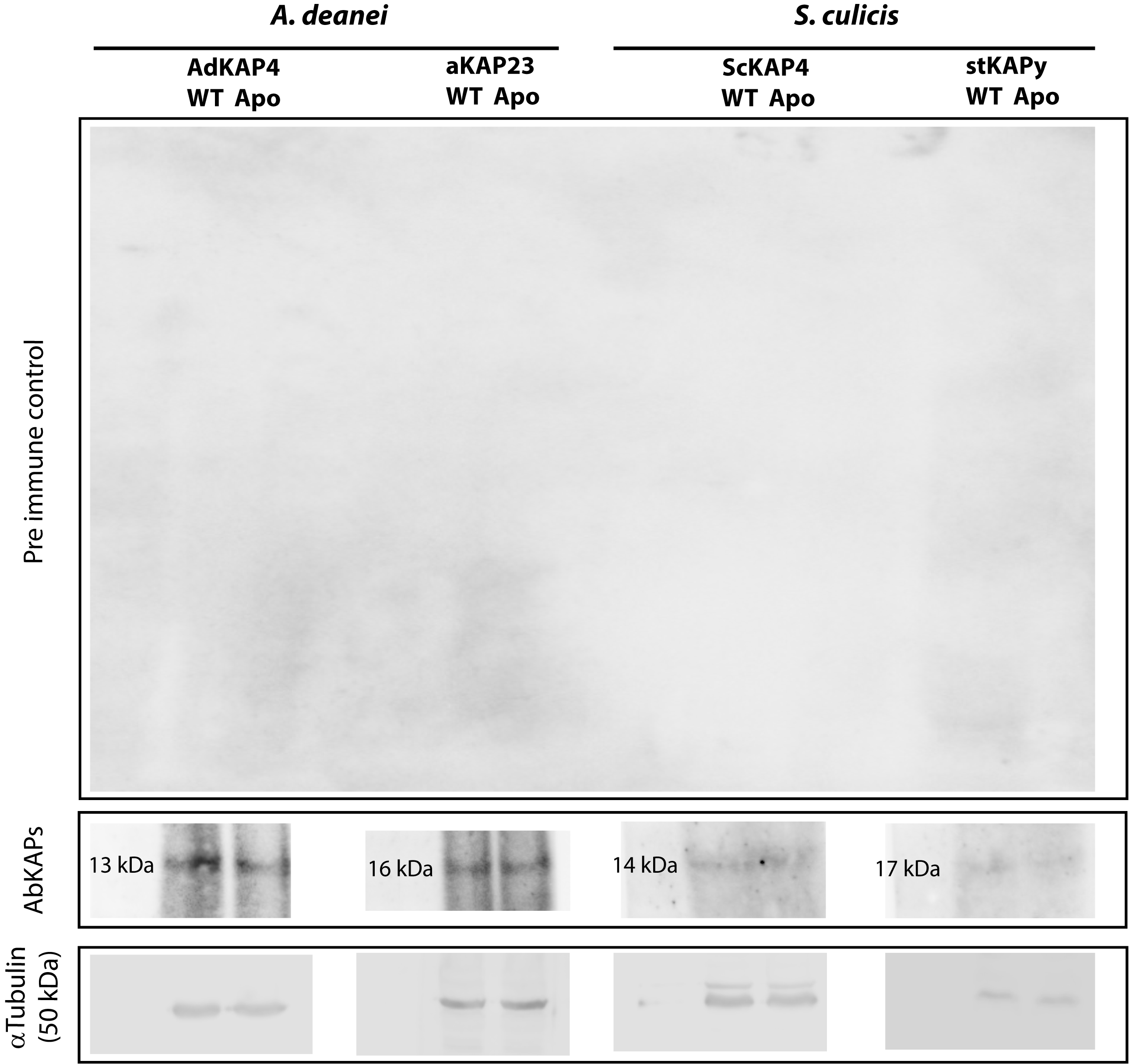

Supplement: S1 Fig — Pre-immune IgG (top, controls); affinity purified IgG antibodies identifying each of the cognate KAP protein (center); Mab antibody to α-tubulin (bottom). (TIF) [file pone.0187516.s001.tif]
