# Supplementary material for: Expanded repertoire of kinetoplast associated proteins and unique mitochondrial DNA arrangement of symbiont-bearing trypanosomatids
Source: PLoS One. 2017 Nov 13;12(11):e0187516. doi: 10.1371/journal.pone.0187516 (PMC5683618; doi:10.1371/journal.pone.0187516)
Supplement: S1 Table — (DOC) [file pone.0187516.s002.doc]

**Supplementary Table 1 – Primers for KAP transcripts quantification by qPCR**

| ***A. deanei* KAPs** | **Primers** |
| --- | --- |
| aKAP23-F | 5' ACC AGA AGT TCC GCA AGC A 3' |
|
| aKAP23-R | 5' GCC GCG ACC CTC TTG TTA G 3' |
|
| KAP4-F | 5'CCC CGC TGA GAA GGA AAA G 3' |
|
| KAP4-R | 5'TGG CGG GCT TCT TGG A 3' |
|
| GADPH-F | 5' GCG TGA ACC AGC AGG AGT ACA 3' |
|
| GADPH-R | 5' AAG GCA GTT GGT GGT GCA A 3' |
|
| ***S. culicis* KAPs** | **Primers** |
| stKAPy -F | 5' CAC CGT CCG CAA GTT GTT C 3' |
|
| stKAPy -R | 5' GGA AAC CCT TGC CCT TCA C 3' |
|
| KAP4-F | 5' GAC CGC GGA AGG ATG CT 3' |
|
| KAP4-R | 5' GCA CAT CGA GCT TCT GCT TCT 3' |
|
|
| GADPH-F | 5' CGG TGC CAA GAC CAT TGT G 3' |
|
| GADPH-R | 5' GCG TTG GAC ACC ACG TTG T 3' |
|
