# Supplementary material for: Expanded repertoire of kinetoplast associated proteins and unique mitochondrial DNA arrangement of symbiont-bearing trypanosomatids
Source: PLoS One. 2017 Nov 13;12(11):e0187516. doi: 10.1371/journal.pone.0187516 (PMC5683618; doi:10.1371/journal.pone.0187516)
Supplement: S2 Table — (DOC) [file pone.0187516.s003.doc]

**Supplementary Table 2 – Taxonomic distribution of putative KAP types among the Trypanosomatidae**

|  | **KAP1** | **KAP2** | **KAP3** | **KAP4** | **KAP6** | **KAP7** | **aKAP23** | **adesKAP** | **stKAPx** | **stKAPw** | **stKAPy** | **stKAPz** | **pKAP** |
| --- | --- | --- | --- | --- | --- | --- | --- | --- | --- | --- | --- | --- | --- |
| *Angomonas deanei* |  |  | X | X | X | X | X |  |  |  |  |  |  |
| *Angomonas desouzai* |  |  | X | X | X | X | X | X |  |  |  |  |  |
| *Strigomonas culicis* |  |  |  | X |  | X |  |  | X | X | X | X |  |
| *Strigomonas galati* |  |  |  | X |  | X |  |  | X | X | X | X |  |
| *Strigomonas oncopelti* |  |  |  | X |  | X |  |  | X | X | X | X |  |
| *Crithidia fasciculata* | X | X | X | X | X | X |  |  |  |  |  |  |  |
| *Leishmania major* | X | X | X | X | X | X |  |  |  |  |  |  |  |
| *Leptomonas seymouri* | X | X | X | X | X | X |  |  |  |  |  |  |  |
| *Phytomonas* sp. EM1 |  |  | X | X | X | X |  |  |  |  |  |  | X |
| *Herpetomonas muscarum* |  |  | X | X | X | X |  |  |  |  |  |  | X |
| *Trypanosoma brucei* |  |  | X^*^ | X | X | X |  |  |  |  |  |  |  |

Notes: An X represents the presence of the KAP clade in an organism; Organisms listed are those present in the phylogenetic tree; The genera *Leishmania*, *Crithidia*, *Leptomonas*, and *Trypanosoma* are represented by just one of the species present in the analysis; *: the KAP3 type is present in two different clades, with the first clade containing exclusively *Trypanosoma* sp. and the second one not containing any *Trypanosoma* sp. sequences.
